# Optical and spectroscopic study of a supersonic flowing helium plasma: energy transport in the afterglow

**DOI:** 10.1038/s41598-020-61988-y

**Published:** 2020-03-20

**Authors:** F. Brandi, L. Labate, D. Rapagnani, R. Buompane, A. di Leva, L. Gialanella, L. A. Gizzi

**Affiliations:** 1 Intense Laser Irradiation Laboratory (ILIL), Istituto Nazionale di Ottica - Consiglio Nazionale delle Ricerche (INO-CNR), Sede Secondaria di Pisa, Via Moruzzi, 1, 56124 Pisa, Italy; 2 Istituto Nazionale di Fisica Nucleare (INFN), Sezione di Pisa, Largo Bruno Pontecorvo, 3, 56127 Pisa, Italy; 3 Dipartimento di Fisica e Geologia, Universitá degli Studi di Perugia, via A.Pascoli, 06123 Perugia, Italy; 4 INFN sezione di Perugia, via A.Pascoli, 06123 Perugia, Italy; 5 Dipartimento di Matematica e Fisica, Universitá della Campania ”L. Vanvitelli”, Viale Lincoln, 5 Caserta, Italy; 6 Dipartimento di Fisica ”E. Pancini”, Universitá di Napoli ”Federico II”, Via Cinthia snc, Napoli, Italy; 7 Istituto Nazionale di Fisica Nucleare, Sezione di Napoli, Via Cinthia snc, Napoli, Italy

**Keywords:** Plasma physics, Techniques and instrumentation

## Abstract

Flowing plasma jets are increasingly investigated and used for surface treatments, including biological matter, and as soft ionization sources for mass spectrometry. They have the characteristic capability to transport energy from the plasma excitation region to the flowing afterglow, and therefore to a distant application surface, in a controlled manner. The ability to transport and deposit energy into a specimen is related to the actual energy transport mechanism. In case of a flowing helium plasma, the energy in the flowing afterglow may be carried by metastable helium atoms and long-lived helium dimer ions. In this work a systematic investigation of the optical and spectroscopic characteristics of a supersonic flowing helium plasma in vacuum and its afterglow as function of the helium gas density is presented. The experimental data are compared with numerical modeling of the plasma excitation and helium dimer ion formation supported by a Computational Fluid Dynamic simulation of the helium jet. The results indicate that the plasma afterglow is effectively due to helium dimer ions recombination via a three-body reaction.

## Introduction

The study of noble gas flowing plasma has gained much attention in recent years due to its impact on the application of cold plasma jet and torches for soft ionization and mass spectrometry^1–10^.

For a flowing helium plasma the dynamics of the energy transport in the afterglow is very complicated due to the peculiar helium plasma characteristics: presence of highly energetic metastable states and energetic excited states close to the ionization limit, as well as the prominent tendency, at increasing particle density, to form helium dimer ions^11,12^. The actual mechanisms and reactions governing the energy and charge transport from the plasma excitation region to the subsequent stream is of importance to understand and optimize the flowing plasma jets performances. Flowing plasma jets are characterized and optimized by playing on the gas flow rate, the plasma excitation power, the distance to the sample to be probed and the nozzle shape^13–15^.

There are two main mechanisms supposedly underlying the energy transport in flowing helium plasma jets and the subsequent reactive nitrogen ion formation in ambient air.

One mechanism is related to the high excited state energy of metastable helium atoms (more than 20 eV) which are formed during the plasma excitation, eventually from cascade transitions from higher excited states. The helium metastable atoms, *He*^*m*^, have long lifetime and therefore, in principle, can live in the afterglow, giving rise eventually to nitrogen ion formation in air through de-excitation and penning ionization in air^11^,

1 $$H{e}^{m}+{N}_{2}\to {N}_{2}^{+}+He+{e}^{-}+\Delta {E}_{1}.$$

On the other side, at increasing particle number density, the excited helium atoms and the helium ions are rapidly and efficiently transformed into helium dimer ions, $H{e}_{2}^{+}$, which have a long lifetime, since they do not posses a permanent dipole moment^12^. In such case the nitrogen ion formation in air is related to dissociation and charge transfer reactions^12^,

2 $$H{e}_{2}^{+}+{N}_{2}\to {N}_{2}^{+}+2He+\Delta {E}_{2}.$$

It is noted that helium ions are rapidly and efficiently converted into helium dimer ion during plasma excitation, and therefore provide very little contribution to the energy transport in the plasma afterglow. Electrons and photons also do not contribute effectively to the energy transport in the afterglow having much lower energy compared to the internal energy of both metastable helium atoms and helium dimer ions.

In order to investigate the flowing helium plasma independently from the interaction with ambient air, and gain insight on the actual flowing plasma dynamics, a systematic optical and spectroscopic investigation of a continuous flowing helium plasma in vacuum was performed as a function of the helium particle density. The plasma is excited by high energy carbon ion bombardment (*C*^3+^, 6 MeV) perpendicular to the He flow direction. Such a configuration basically projects in space the time dependent processes of the helium plasma excitation and termination, thus allowing the use of optical imaging and spectroscopy to follow the system dynamics at the hundreds of ns time scale. Moreover, the flowing configuration is similar to the actual design used in plasma pencil and jets^3,9,16^.

## Results

**Figure 1:**
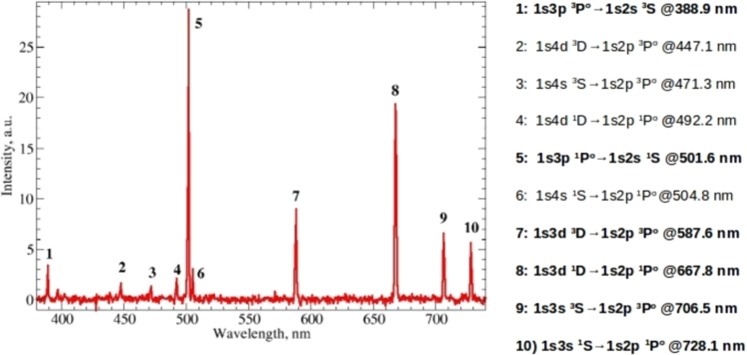
Optical Emission Spectroscopy of the He plasma: the graph shows the typical overall emission spectrum of the supersonic helium jet with a density of 3.9 × 10^18^ cm^−3^ (see Methods and Supplementary Information) when interacting with the carbon ion beam. The assignment of the main lines is reported on the right side, with the most intense lines highlighted in bold.

Figure 1 shows the time integrated spectrum of the overall optical emission from the helium plasma jet in vacuum excited by a 6 MeV carbon ion beam. All emission lines are assigned to helium excited states^17^. The basic processes for plasma excitation by ion bombardment are impact ionization and excitation, leading to the formation of helium ion *He*^+^ and excited helium atoms He^*^ respectively. The cross section for excitation and ionization, obtained interpolating known values at 11 MeV^18^ and 2.4 MeV^19^, are of the same order of magnitude, *σ*(*C*^3+^) ~ 10^−17^ cm^2^, therefore the density of ionized and excited helium is about the same during plasma formation by ion bombardment. The actual rate coefficient for these reactions is given by *σ*(*C*^3+^)*V*(*C*^3+^) = 9.8 × 10^−9^ cm^3^s^−1^, where *V*(*C*^3+^) ~ 9.8 × 10^8^ cm s^−1^ is the carbon ion velocity.

**Figure 2:**
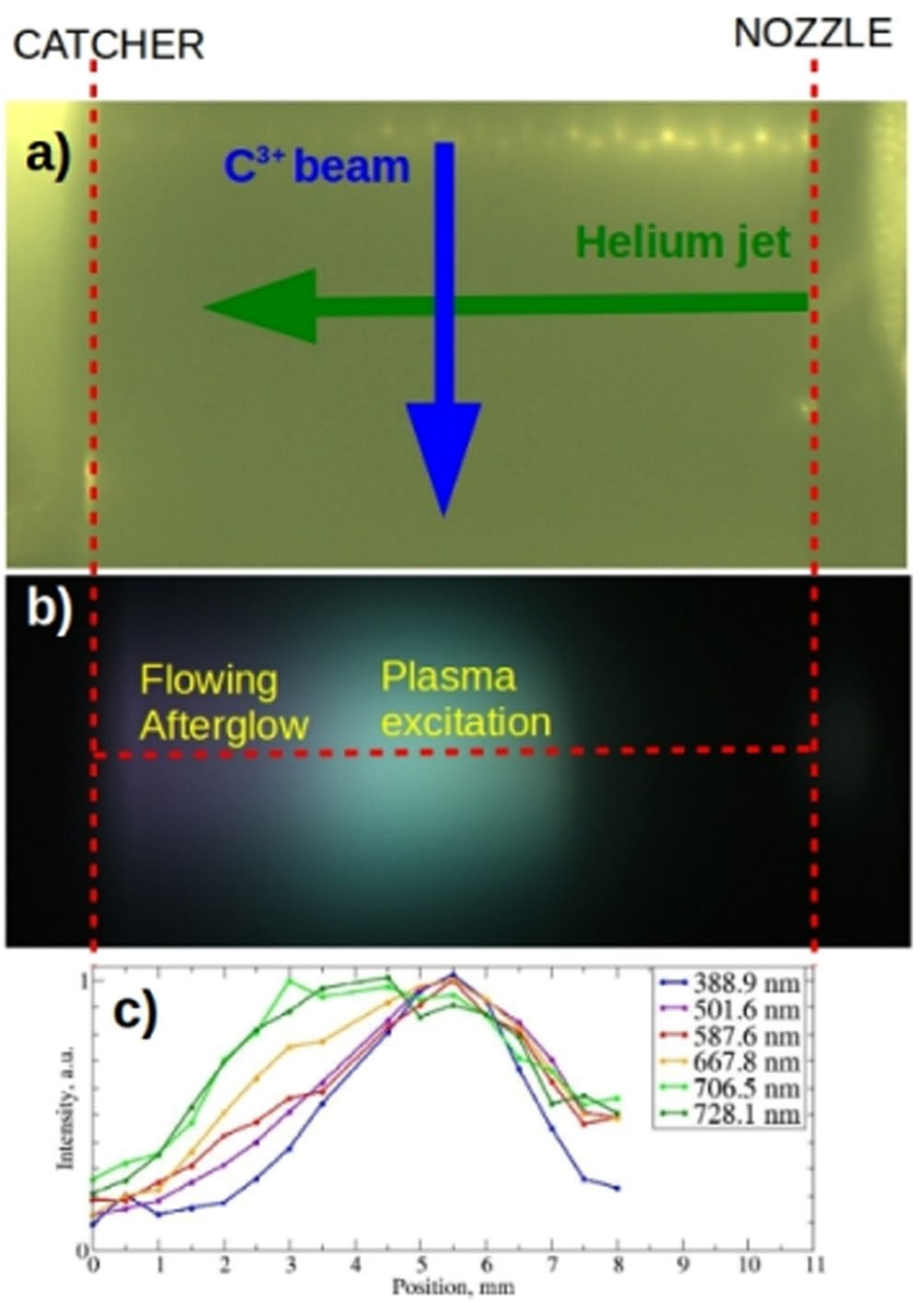
Spatially resolved Optical Emission Spectroscopy. (**a**) Image of the interaction region with the nozzle on the right the catcher on the left (positions indicated by the vertical dashed red lines). (**b**) Image of the plasma formed by the interaction of the carbon ion beam and the supersonic helium jet with a density of 3.9 × 10^18^ cm^−3^. (**c**) Spatially resolved spectroscopy of the six most intense helium emission lines along the horizontal dashed red line shown in (**b**), where each curve is normalized to its maximum value for better comparison.

In Fig. 2(a,b) the optical images of the interaction region are reported. Figure 2(a) shows the interaction region without plasma when illuminated from outside the vacuum chamber: the nozzle and the catcher are visible on the right and on the left of the image respectively (position indicated by vertical dashed lines), with He flowing from right to left, while the spacing screw is visible on the top of the image (see Supplementary Fig. S1 for a panoramic view of the nozzle assembly). The catcher is placed at 11 mm from the nozzle.

Figure 2(b) shows the image of the few mm in diameter uncollimated carbon ion beam interacting with the helium jet at a density of 3.9  × 10^18^ cm^−3^. The velocity of the helium jet is estimated computationally to be ~1700 m/s (see Methods and Supplementary Fig. S2), therefore 1 mm in the flowing direction corresponds to 590 ns in time-domain. The round bright area indicates the plasma excitation region where the uncollimated carbon ion beam crosses the helium jet, while a flowing afterglow is clearly visible on the left, towards the catcher. It must be noted that the “violet” color of the afterglow is due to a combination of the spectral distribution of the emission lines from the plasma afterglow and the response of the color camera which has non negligible sensitivity to near-infrared radiation in the blue-channel.

Spatially resolved Optical Emission Spectroscopy (OES) indicates a distinct emission between the plasma excited in the interaction region, at ~5.5 mm from the catcher and the subsequent afterglow (see Methods for experimental details). In Fig. 2(c) the intensity of the six main He emission lines is reported as function of the longitudinal position. Basically, all lines are emitted from the plasma excitation region, while transitions from 3s and 3d excited states are mainly present in the afterglow. All these helium energy levels however have a short life time (less then hundreds of ns^20^), so they are not expected to survive in the afterglow, i.e., several *μ*s after leaving the plasma excitation region. Instead, excited helium in the 3s and 3d energy levels predominantly present in the afterglow may arise from the recombination of He dimer ions in the afterglow^21,22^.

**Figure 3:**
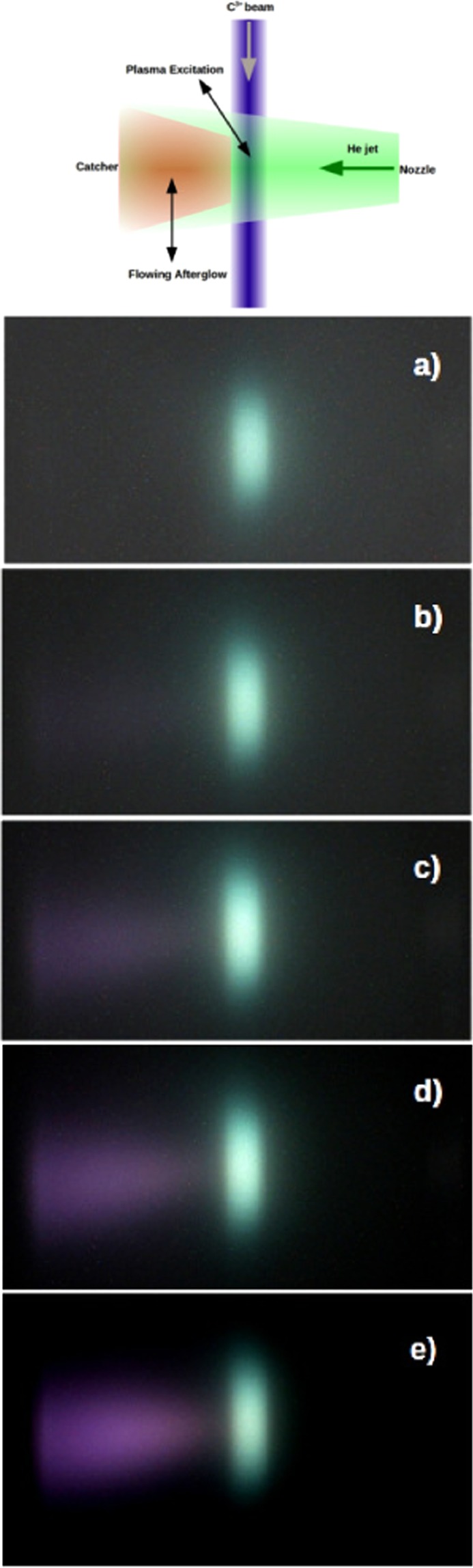
Imaging of the flowing helium plasma excited by the collimated ion beam. Upper image: schematic representation of the interaction between the collimated carbon ion beam and the helium jet. Images of the flowing plasma as function of gas density (in 10^18^ cm^−3^): (**a**) 2.3, (**b**) 3.4, (**c**) 3.9, (**d**) 4.6, (**e**) 5.0. The contrast/luminosity of the images have been altered to highlight the qualitative characteristics of the flowing afterglow.

To get further insight on the afterglow light emission, a systematic investigation of the helium plasma and its afterglow was performed as function of the helium jet density. In order to have a higher space/time resolution of the plasma and afterglow evolution, the carbon ion beam is collimated to 1 mm in its central part using a hard mask on the beam. In the upper panel of Fig. 3 a schematic view of the interaction between the collimated ion beam and the helium jet is shown. In Fig. 3(a–e) the images of the plasma and its afterglow with increasing helium density are shown. The plasma excitation region is clearly evident as a pale green coloured, cigar shaped region, that reflects the 1 mm in diameter carbon ion beam interacting with the few mm wide supersonic helium beam. As the helium density increases from 2.3 × 10^18^ cm^−3^ to 5.0 × 10^18^ cm^−3^ (see Methods and Supplementary Fig. S3), a clear afterglow appears, whose intensity has a highly non-linear dependence on the helium gas density.

**Figure 4:**
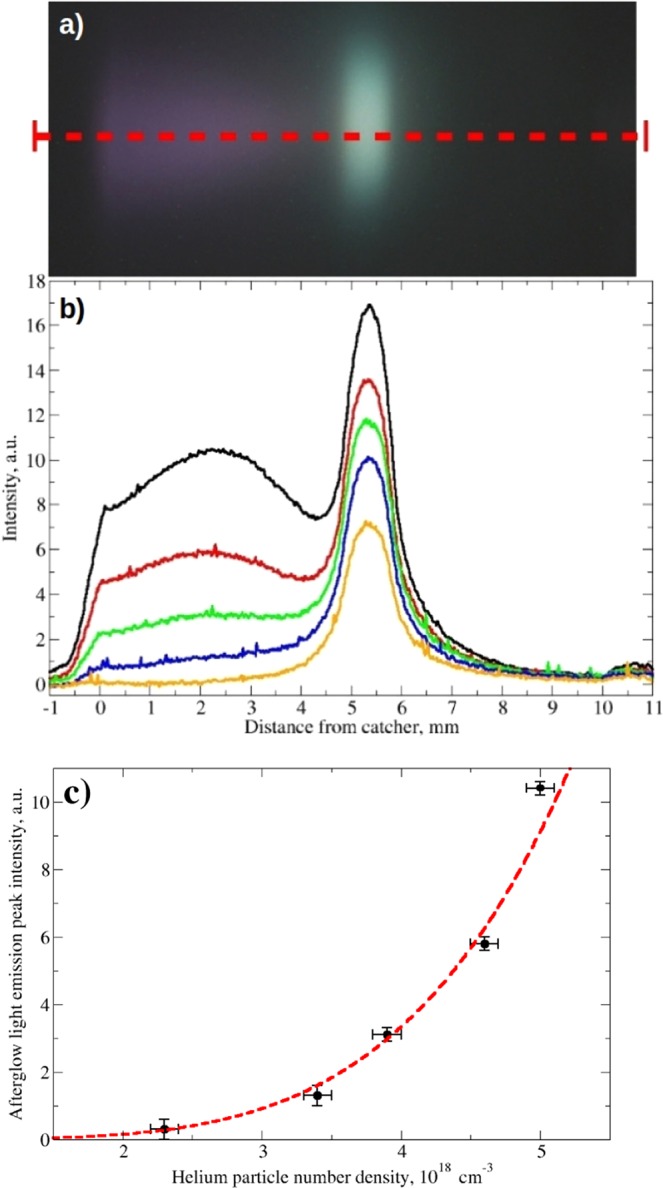
Afterglow light emission analysis. (**a**) The dashed red line indicates the longitudinal line-out considered for the data analysis. (**b**) Intensity of the plasma and afterglow as function of helium gas density, from bottom to top the curves correspond to a peak density of 2.3, 3.4, 3.9, 4.6, 5.0 in units of 10^18^ cm^−3^ respectively, as from images in Fig. 3. (**c**) Evolution of the afterglow peak emission value as function of helium gas density; the red dashed curve is the result of a two-parameter single power function fit, *y* = *A* × *x*^*B*^.

In Fig. 4, both the line-out of the light emission intensity along the gas-jet propagation axis, as highlighted in Fig. 4(a), and the afterglow peak emission intensity *vs* the peak gas density are reported (Fig. 4(b,c) respectively).

The most striking feature of the optical imaging data is the clear appearance with increasing helium density of a local minimum in the light emission right after the excitation region and before the afterglow. Given the effect of the plasma motion, this minimum in space corresponds to a minimum in time at a few 100s of ns after the plasma excitation.

Two results are evident: (i) the linear increase of the light emission intensity within the plasma excitation region (see Supplementary Figs. S4 and S5); (ii) the highly non-linear increase of the light emission intensity in the afterglow as function of the He density.

A least-square fit of the experimental data points of the peak value of the afterglow light emission profile *vs* helium density, using a two free-parameter power function, i.e., *y* = *A* × *x*^*B*^, results in a power coefficient of B = 4.5(5), as shown in 4(c).

**Figure 5:**
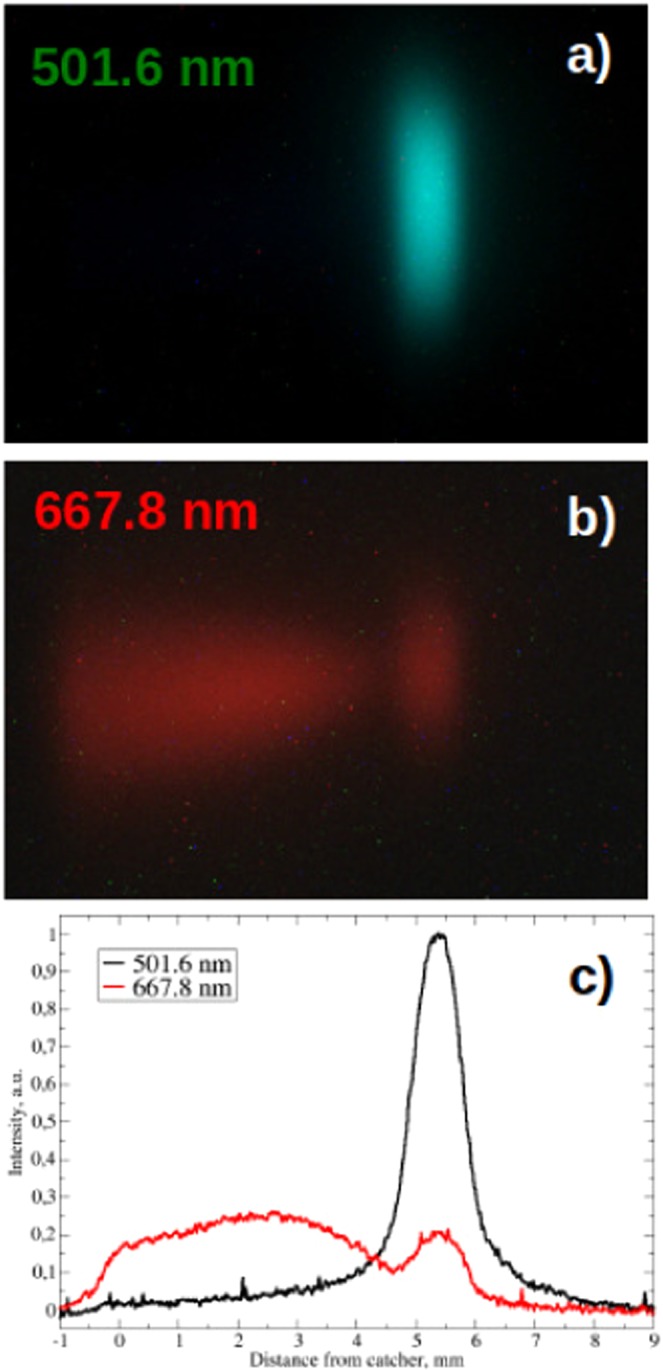
Spectral imaging of the plasma. (**a**) Spatially resolved emission of the 501.6 nm helium line. (**b**) Spatially resolved emission of the 667.8 nm helium line. (**c**) Intensity of the two emission lines along the longitudinal line-out. The contrast/luminosity of the images have been altered to highlight the qualitative characteristics of the flowing afterglow.

Spectral imaging of the helium jet interacting with the collimated carbon ion beam confirms the striking difference in the light emission spectrum between the plasma excitation region and the flowing afterglow. Figure 5(a,b) show the images of the light emission at 501.6 nm (1*s*3*p*^1^*P*^*o*^ → 1*s*2*s*^1^*S*) and 667.8 nm (1*s*3*d*^1^*D* → 1*s*2*p*^1^*P*^*o*^), respectively. The intensity profile of the two images along the central longitudinal line-out are compared in Fig. 5(c). The emission of the 501.6 nm line is substantially limited into the plasma excitation area, while the emission of the 667.8 nm line extends significantly in the afterglow. The emission from the 3d He excited state in the afterglow is an indication of He${}_{2}^{+}$ recombination^21,22^.

## Discussion

During plasma formation, He^+^ and He^*^ created by C^3+^ impact excitation and ionization of helium atoms transform rapidly into helium dimer ion, in fact He${}_{2}^{+}$ is well known to be the most abundant ion species in medium/high density helium plasma^12,23,24^. Helium dimer ions formation follows fundamentally two paths:

(i) Three-body association reaction,
  3 $$H{e}^{+}+2He\to H{e}_{2}^{+}+He,$$
  with a rate constant of 1.3 × 10^−31^ cm^6^s^−1^ ^12,23^;
(ii) Associative ionization,
  4 $$H{e}^{\ast }(n\ge 3)+He\to H{e}_{2}^{+}+{e}^{-},$$
  with a rate constant in between 0.13 to 8.2 × 10^−10^ cm^3^ s^−1^,^12,23,24^. Several investigations have indicated that associative ionization is a nearly resonant reaction with the 3d energy level, resulting in molecular ions in a vibrational excited state (*ν* = 4)^25^. This is consistent with the observation of low emission of the 667.8 nm line in the plasma excitation region since the helium atoms excited in the 3d level will rapidly transform into helium dimer ions quenching the light emission.

To elucidate the contributions of the various reactions involved a comprehensive numerical calculation including the rate equations of the reactions present during plasma excitation (i.e., creation of He^+^ and He^*^ by carbon ion bombardment) and helium dimer ion formation (i.e., Eq. (3) and Eq. (4)) is performed (see Methods and Supplementary Fig. S7). From this calculation it results that the helium dimer ion formation reactions are very fast in the present experimental conditions, such to effectively saturate He^+^ and He^*^ created by carbon ion bombardment. As a consequence the electron density has an effective linear dependence on the helium density, i.e., [e] ∝ [He] (see Supplementary Fig. S8), while the density of helium dimer ion as function of the helium density [He] follows effectively a power law with power coefficient 1.56(4) (see Supplementary Fig. S8).

The appearance of a flowing afterglow with a dip in light emission followed by a maximum, as shown in Fig. 3, is in close resemblance of the data reported on the spatially resolved spectroscopic investigation in a flowing helium plasma based ionization source in ambient air^12,26^. A distinct afterglow very sensitive to He pressure, ascribed to He${}_{2}^{+}$ recombination, has been observed in a microwave excited flowing helium plasma in vacuum^27^. A high resolution time dependent investigation of the light emission in a helium plasma afterglow also revealed the presence of a local minimum or “dark gap” in the helium afterglow light emission^28^.

In order to interpret the results obtained by the optical investigation of the supersonic flowing helium plasma, the origin of light emission in the afterglow is analyzed. In general, the light emission in the afterglow may arise from:

1. He^*^ created during plasma excitation. This source can be ruled out since the life time of helium excited states is less then hundreds of ns, and it would induce a monotonically decreasing light emission out-side the plasma excitation region.
2. He^*^ created by stepwise electron excitation of metastable helium atoms. The cross section for excitation to the triplet 3p state is two times larger then the cross section for excitation to the triplet 3s state^29^. The OES measurements, see Fig. 2, show a prominent contribution from the triplet 3s state in the afterglow with negligible contribution from the triplet 3p state. Therefore stepwise excitation of metastable helium atoms can be ruled out as the source for the strong light emission in the plasma afterglow.
3. He^*^ created during the recombination of the long-living ion species He${}_{2}^{+}$. This scenario is supported by the spatially resolved spectroscopic measurements, showing emission from 3d and 3s He excited states in the afterglow.

Therefore light emission in the afterglow is due to recombination processes. Two possible He${}_{2}^{+}$ recombination routes can be considered:

(i) direct ion-electron dissociative recombination according to the following reaction,
  5 $$H{e}_{2}^{+}+{e}^{-}\to H{e}^{\ast }+He.$$
  In this case, [He^*^]  ∝  [He${}_{2}^{+}$][e]  ∝  [He]^2.6^, where the last relations follows from the computed rate equations during plasma excitation and helium dimer ion formation.
(ii) three-body reaction to form an intermediate highly excited He Rydberg dimer^30^, He${}_{2}^{\ast \ast }$,
  6 $$H{e}_{2}^{+}+{e}^{-}+{e}^{-}(He)\to H{e}_{2}^{\ast \ast }+{e}^{-}(He),$$
  followed by collisional dissociation,
  7 $$H{e}_{2}^{\ast \ast }+He\to H{e}^{\ast }+2He.$$
  In this case, [He^*^] ∝ [He${}_{2}^{* * }$][He]  ∝  [He${}_{2}^{+}$][He]^3^ ∝  [He]^4.6^, where the last relation follows from the computed rate equations during plasma excitation and helium dimer ion formation.

The experimental results show a power coefficient of 4.5(5) for the plasma afterglow emission intensity as function of the helium jet density, see Fig. 4, which indicates that three-body reaction followed by collisional dissociation is dominant in the density range studied.

The presented approach provides a relatively simple methodology to study flowing plasma jets and determine the dominant reaction chain ruling the energy transport in the afterglow. In perspective, experiments at higher gas density will allow to investigate the onset of other paths for active spices formation during plasma excitation, as well as for recombination in the afterglow. At higher density values for example the trimer ion He${}_{3}^{+}$ can be produced in the plasma, whose recombination rate would exceeds that of He${}_{2}^{+}$ in the afterglow^31^. Also, increasing the neutral gas density the formation of He${}_{2}^{+}$ would start to follow predominantly the three-body reaction, reported in Eq. (3), rather then the associative ionization reported in Eq. (4). Therefore a different power law governing light emission as function of gas density is expected when increasing the neutral gas density in the flowing jet. In summary, a systematic optical imaging and emission spectroscopy study of a supersonic helium plasma jet has been performed as function of the gas density. A striking visible flowing afterglow separated from the plasma excitation region is found, with the emission intensity increasing highly nonlinearly with the helium density. A comprehensive numerical calculation of the rate equations governing the plasma excitation and the helium dimer ion formation and a CFD simulation on the surpesonic jet expansion in vacuum have been performed to analyse the experimental data. After a quantitative analysis of the afterglow optical emission it can be concluded that helium dimer ion formation, transport and recombination, through an intermediate highly excited He Rydberg dimer molecule followed by collisional dissociation, is the prevalent energy/charge transport process in the afterglow of a helium flowing plasma in the density range studied.

The optical study of flowing plasma in vacuum is found to be an efficient tool to investigate in details the evolution of the plasma from excitation to termination. Experiments at higher neutral gas density and the use of a controlled introduction of N_2_/O_2_ gas down-stream the plasma will provide further insight on the actual plasma afterglow-air interaction.

## Methods

### The plasma jet

The continuous flowing helium plasma jet is excited by a continuous carbon ion beam from the TANDEM accelerator at the CIRCE DMF University of Campania (Italy). The ion beam consists of 6 MeV C^3+^ ions and it is few mm wide. A collimated carbon ion beam with a diameter of 1 mm is obtained using a hard aperture, i.e., a beam-dump comprising a 1 mm hole, which is positioned in vacuum few cm before the helium supersonic jet. The current of the collimated carbon beam is 100nA. The continuous supersonic helium jet^32^ is expanding in vacuum perpendicular to the carbon beam and it is produced by a valve composed of a nozzle and a catcher. The nozzle has a De Laval profile with an exit diameter of 2 mm. The catcher is placed 11 mm from the nozzle. An image of the valve assembly in vacuum is shown in Supplementary Fig. S1. The carbon ion beam intersects the helium jet at 5.5 mm from the nozzle. The valve is fed with high purity helium (99.999%) at a variable backing pressure, while the flow rate is measured with a digital flow-meter. The helium gas flow rate to the nozzle is linear as function of backing pressure (see Supplementary Fig. S5). The helium jet expands in the interaction chamber with 10^−2^ mbar background pressure. The interaction chamber is constantly pumped by means of turbomolecular pumps, and the background gas is mainly residual helium. During the measurements the helium is let to continuously flow in the chamber and the dynamic pressure in the chamber rises to about 10^−1^ mbar; no significant impact on the measurements is expected from the impurities in the vacuum chamber. The jet velocity on the valve axis is computed to be 1700 m/s with less then 10% variation in the accessible pressure range from 4 to 9 bar (see Supplementary Fig. S2). The helium gas density along the axis of the jet is evaluated by Computational Fluid Dynamic simulations (see Supplementary Fig. S3), and its value at the crossing with the carbon ion beam vary between 2.3  × 10^18^ cm^−3^ to 5.0  × 10^18^ cm^−3^ in the baking pressure range from from 4 to 9 bar.

### Optical imaging

The interaction region is imaged with magnification 0.5 into a color camera (Basler, acA2000-50gc) by a 10 cm focal length achromatic doublet lens. The camera line of sight is set radially to the helium jet axis, at 45 degrees with respect to the carbon beam. For comparative analysis the data from the images are normalized to 1.5 sec acquisition time.

The transverse line-out of the light emission from the plasma excitation region shows a Gaussian profile (see Supplementary Fig. S4), with a FWHM of 4 mm at backing pressures higher than 7 bar, that reduces to 3.5 mm at 4 bar (see Supplementary Fig. S5).

Spectral imaging reported in Fig. 5(a,b) is performed using 2 band-pass filters in front of the camera, centered respectively at 500 nm and 650 nm, both with 40 nm pass band. The intensity of the camera images are set by adjusting the integration time.

### Optical emission spectroscopy

Using the same optical imaging set-up, optical emission spectroscopy is performed with a fiber-coupled spectrometer (Ocean Optics HR2000), in combination with a multi-mode quartz optical fiber with 1 mm diameter. Spatially resolved optical emission spectroscopy is obtained by translating the optical fiber along the longitudinal axis of the helium jet in the image plane. Therefore each data point reported in Fig. 2(c) is the average over a region of 2 mm in diameter in the actual helium jet.

### Numerical modeling

The Computational Fluid Dynamic simulation was performed using ANSYS Fluent 17.1.0 as described in^32^. Summarizing, an axial symmetry of the system is assumed and the problem is solved in 2D. The whole expansion chamber is simulated and the problem is solved imposing as boundary conditions the values of the pressure in the helium tank, in the expansion chamber and in the catcher. A Realizable *k* − *ε* model is used to threat the turbulence, and the problem is evaluated until the second order (pressure-velocity coupled scheme).

In order to gain some insights of the dynamics of the main chemical processes involved in the energy exchange and transfer, a set of rate equations was built up and solved numerically, accounting for the reactions discussed in the text (and listed in the Supplementary Materials). The gas-jet evolution was considered in the rate equations by adding a dissipative term accounting for the gas expansion dynamics as retrieved by the CFD simulation (see Supplementary Fig. S3). The He gas was supposed to interact with a C ions beam exhibiting a transverse lorentzian profile with 1 mm FWHM. The system of ordinary differential equations was solved numerically using a 4^*th*^ order Runge-Kutta method with adaptive stepsize^33^. A few plots showing the results of this modelling are shown in the Supplementary Figs. S7 and S8.

## Supplementary information

Supplementary Information.

## Data Availability

The datasets generated during and/or analysed during the current study are available from the corresponding authors on reasonable request.
