# Supplementary material for: Optical and spectroscopic study of a supersonic flowing helium plasma: energy transport in the afterglow
Source: Sci Rep. 2020 Mar 20;10:5087. doi: 10.1038/s41598-020-61988-y (PMC7083830; doi:10.1038/s41598-020-61988-y)
Supplement: Supplementary file 1 — Supplementary Information. [file 41598_2020_61988_MOESM1_ESM.pdf]

Supplementary Information

Optical and spectroscopic study of a supersonic flowing helium plasma: energy transport in the afterglow

F. Brandi[1,*], L. Labate[1,2,+], D. Rapagnani[3,4,5], R. Buompane[5,7], A. di Leva[6,7], L. Gialanella[5,7], and L. A. Gizzi[1,2]

[1] Intense Laser Irradiation Laboratory (ILIL), Istituto Nazionale di Ottica - Consiglio Nazionale delle Ricerche (INO-CNR), Sede Secondaria di Pisa, Via Moruzzi, 1, 56124 Pisa, Italy.
[2] Istituto Nazionale di Fisica Nucleare (INFN), Sezione di Pisa, Largo Bruno Pontecorvo, 3, 56127 Pisa, Italy.
[3] Dipartimento di Fisica e Geologia, Università degli Studi di Perugia, via A.Pascoli, 06123 Perugia, Italy.
[4] INFN sezione di Perugia, via A.Pascoli, 06123 Perugia, Italy.
[5] Dipartimento di Matematica e Fisica, Università della Campania "L. Vanvitelli", Viale Lincoln, 5, Caserta, Italy.
[6] Dipartimento di Fisica "E. Pancini", Università di Napoli "Federico II", Via Cinthia snc, Napoli, Italy.
[7] Istituto Nazionale di Fisica Nucleare, Sezione di Napoli, Via Cinthia snc, Napoli, Italy.
[*] fernando.brandi@ino.cnr.it
[+]luca.labate@ino.cnr.ii

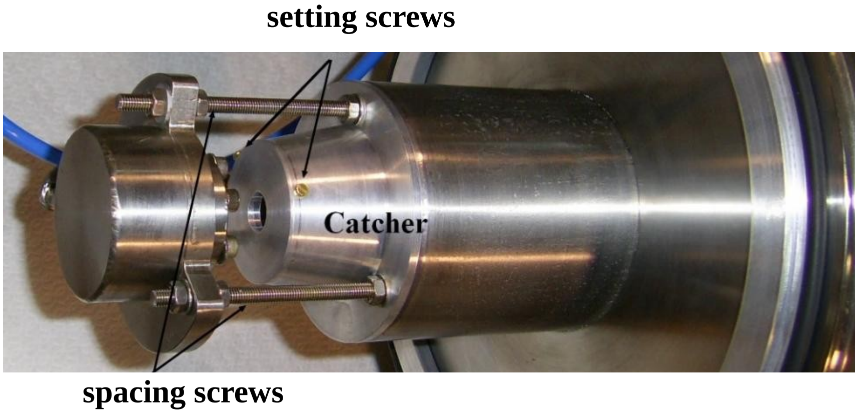

Figure S1. Actual picture of the valve assembly. The nozzle is on the left and the catcher is visible on the right. The setting and spacing screws are indicated.

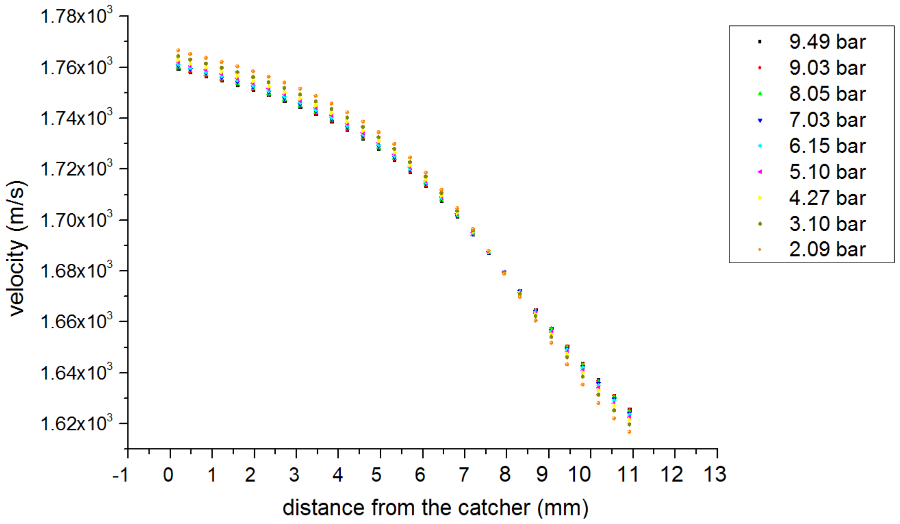

Figure S2. Helium jet velocity. The velocity has been calculated by Computational Fluid Dynamics simulations (ANSYS Fluent software) as function of the gas backing pressure.

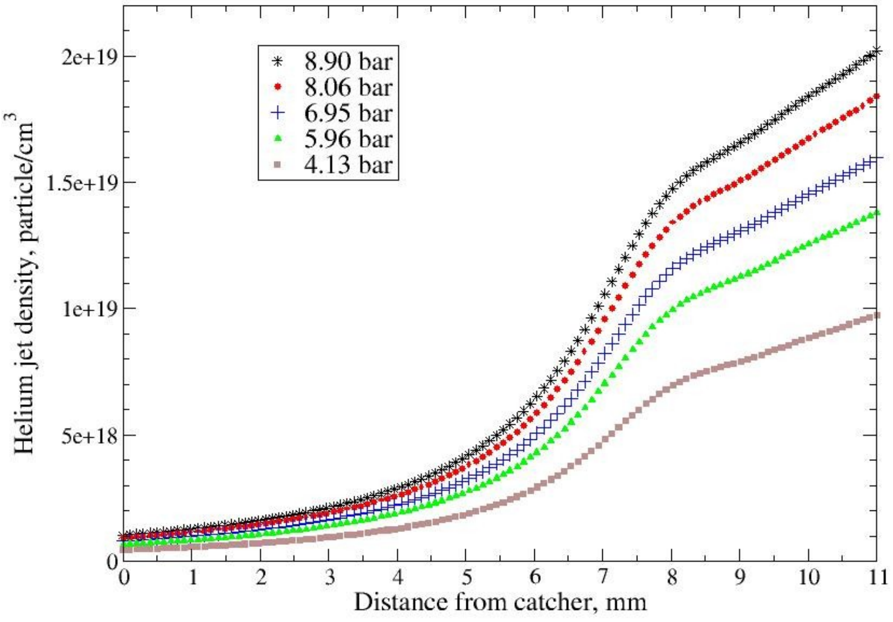

Figure S3. Helium jet density. The particle density has been calculated by Computational Fluid Dynamics simulations (ANSYS Fluent software) as function of the gas backing pressure. The helium jet density at carbon ion beam position are 5.0, 4.6, 3.9, 3.4, 2.3 in units of 10^{18} cm^{-3} for helium backing pressure of 8.90, 8.06, 6.95, 5.96 and 4.13 bar respectively.

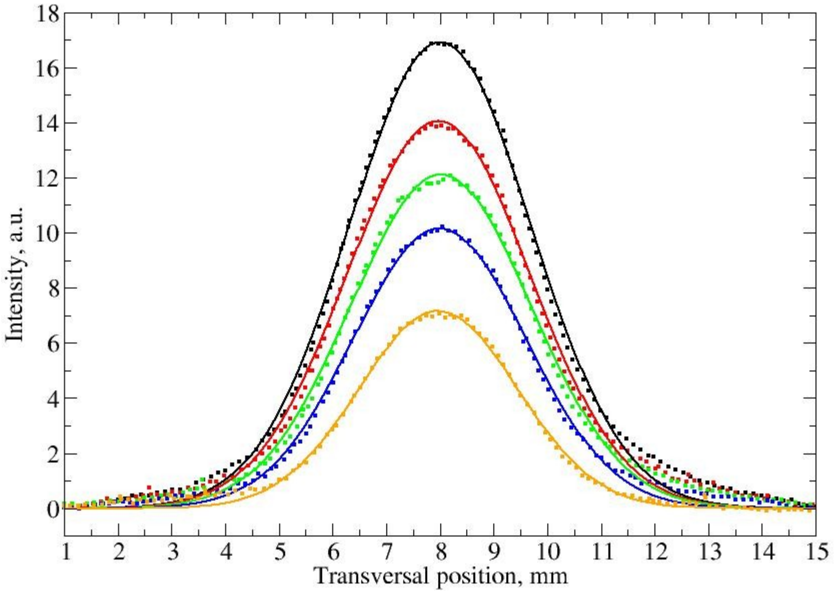

Figure S4. Plot of the transverse cross-section of the light emission from the plasma excitation region as function of helium density. The continuous curves represent the results of Gaussian fit to the experimental data points. From top to bottom the curves correspond to a peak density of 5.0, 4.6, 3.9, 3.4, 2.3 in units of 10^{18} cm^{-3} respectively.

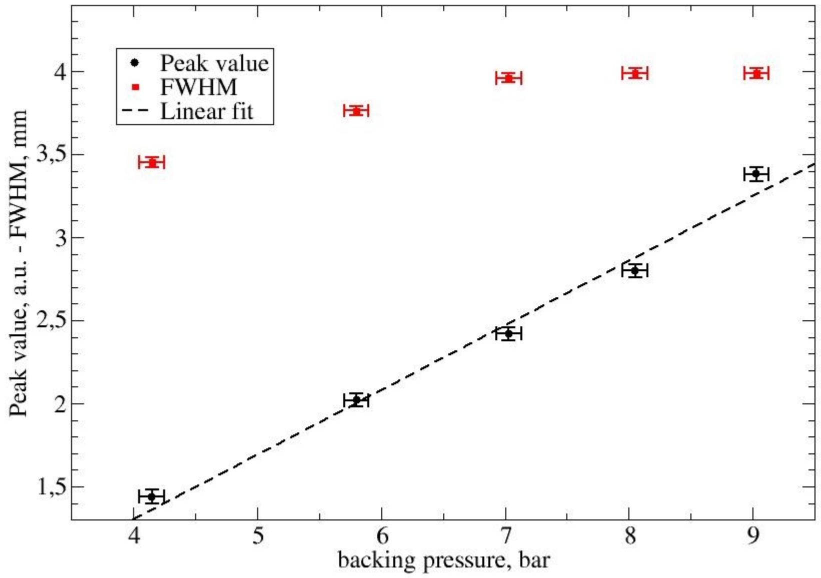

Figure S5. Analysis of the light emission in the plasma excitation region. The peak value and the full-width half maximum (FWHM) obtained from the Gaussian fit of the transverse plasma emission shown in Figure S3. The dashed line represent the result of a linear least-square fit on the experimental peak value data points .

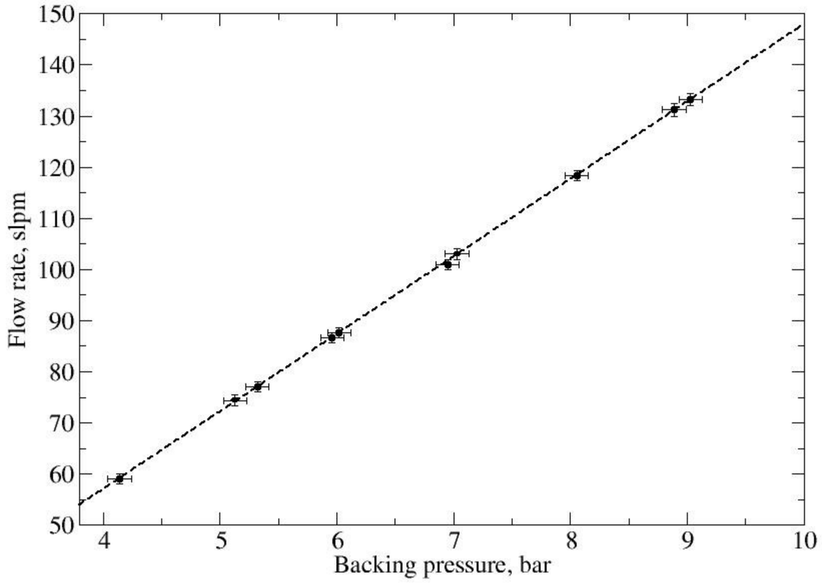

Figure S6. Helium flow rate as function of the gas backing pressure. The result of the linear least-square fit is shown by the dashed line.

Numerical modeling

Numerical calculation is performed to solve the following rate equation system:

$d[He^*]/dt = K_1[C^{3+}][He]$ - Plasma Excitation by Carbon ion bombardment,

$d[He^+]/dt = K_1[C^{3+}][He]$ - Plasma Excitation by Carbon ion bombardment,

$d[He_2^+]/dt = K_2[He^+][He]^2 + K_3[He^*][He]$ - Helium dimer ion formation,

$d[e^-]/dt = K_1[C^{3+}][He] + K_3[He^*][He]$ – Free electron generation,

where $K_1 = 9.8x10^{-9}$ cm^3s^{-1}, $K_2 = 1.3x10^{-31}$ cm^6s^{-1}, $K_3 = 1.3x10^{-11}$ cm^3s^{-1} and $[C^{3+}] = 2.66x10^4$ cm^{-3} at 100 nA ion beam current.

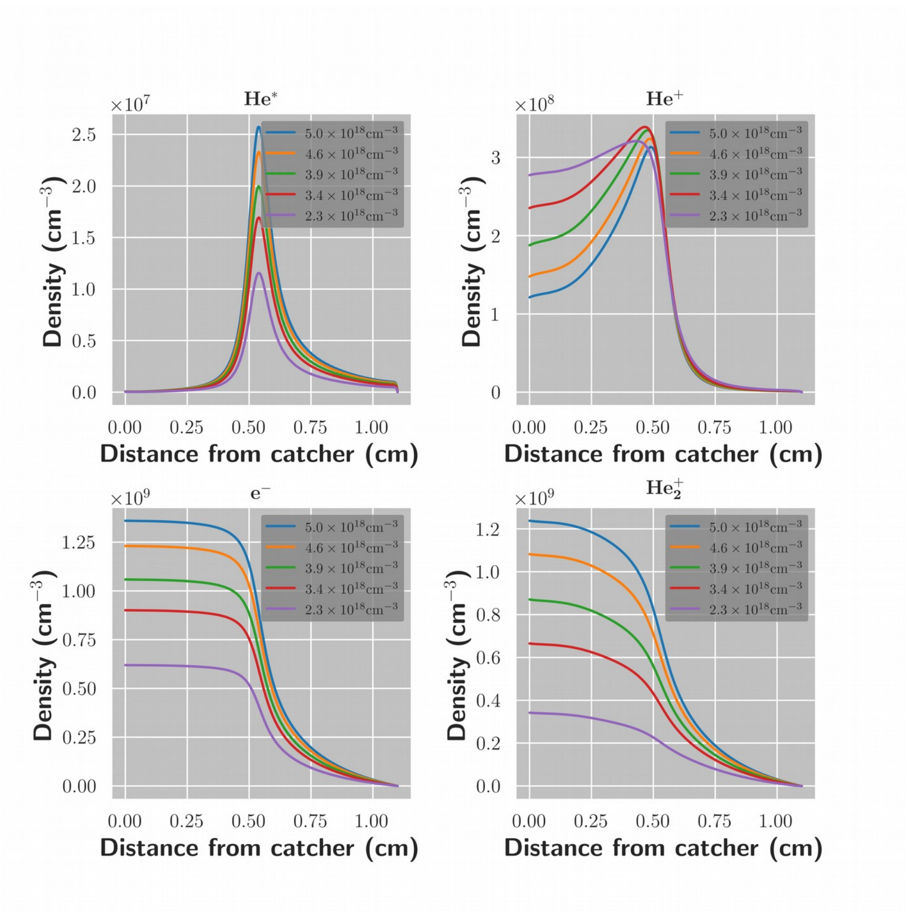

Figure S7. Results of the numerical calculation of the rate equation system reported above. The density is shown as function of the distance from the catcher for various helium jet densities.

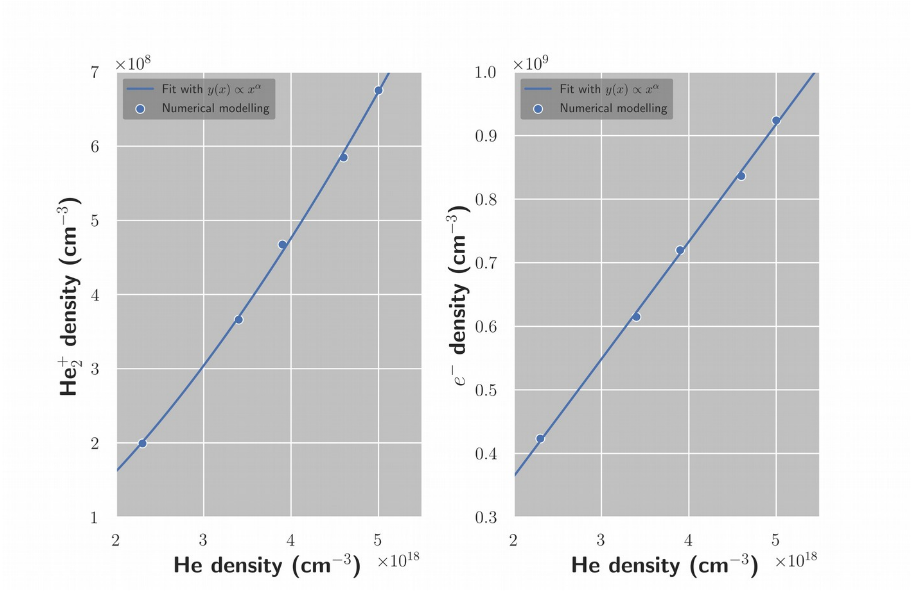

Figure S8. Calculated [He_2^+] and [e^-] as function of the helium density. The dots are the data points resulting from the numerical modeling (see Fig. S7) and the line is the result of a power law fit on the data points; the resulting power coefficients are 1.56(4) and 1.01(2) for He_2^+ and e^- respectively.
